# Complexity Analysis of Electroencephalogram Dynamics in Patients with Parkinson's Disease

**DOI:** 10.1155/2017/8701061

**Published:** 2017-02-20

**Authors:** Guotao Liu, Yanping Zhang, Zhenghui Hu, Xiuquan Du, Wanqing Wu, Chenchu Xu, Xiangyang Wang, Shuo Li

**Affiliations:** ^1^School of Computer Science and Technology, Anhui University, Hefei 230601, China; ^2^Center for Optics and Optoelectronics Research, College of Science, Zhejiang University of Technology, Pingfeng Campus, Liuhe Road 288, Xihu District, Hangzhou 310023, China; ^3^Shenzhen Institutes of Advanced Technology, Chinese Academy of Sciences, Shenzhen 518055, China; ^4^Anhui Electrical Engineering Professional Technique College, Hefei 230051, China; ^5^Department of Medical Imaging, Schulich School of Medicine and Dentistry, University of Western Ontario, 1151 Richmond St, London, ON, Canada

## Abstract

In this study, a new combination scheme has been proposed for detecting Parkinson's disease (PD) from electroencephalogram (EEG) signal recorded from normal subjects and PD patients. The scheme is based on discrete wavelet transform (DWT), sample entropy (SampEn), and the three-way decision model in analysis of EEG signal. The EEG signal is noisy and nonstationary, and, as a consequence, it becomes difficult to distinguish it visually. However, the scheme is a well-established methodology in analysis of EEG signal in three stages. In the first stage, the DWT was applied to acquire the split frequency information; here, we use three-level DWT to decompose EEG signal into approximation and detail coefficients; in this stage, we aim to remove the useless and noise information and acquire the effective information. In the second stage, as the SampEn has advantage in analyzing the EEG signal, we use the approximation coefficient to compute the SampEn values. Finally, we detect the PD patients using three-way decision based on optimal center constructive covering algorithm (O_CCA) with the accuracy about 92.86%. Without DWT as preprocessing step, the detection rate reduces to 88.10%. Overall, the combination scheme we proposed is suitable and efficient in analyzing the EEG signal with higher accuracy.

## 1. Introduction

Parkinson's disease (PD) is a physical disorder that occurs in the brain and affects the motor system. It is the second most common neurodegenerative disease after Alzheimer's disease. 1% to 2% of the elderly are affected because the incidence increases above the age of 50 ‎[1]. Most cases of PD are sporadic and known as idiopathic. However, the causes of PD are unknown; several possible mechanisms have been proposed, such as exogenous toxins, inflammation, genetic mutations, and the combinations of these factors ‎[2]. A generally accepted hypothesis is that PD is related to genetic and environmental factors most. In PD patients, there are three cardinal motor symptoms, such as bradykinesia, rigidity, and tremor. Some statistics about PD indicate that tremor is its most visible signature. Tremor is the predominant symptom in some PD patients, while, in others patients, the tremor is absent or mild. The gait problems are another spectrum of PD symptoms, characterized by shuffling, small steps, decreased arm swing, and a forward bended posture. Furthermore, freezing of gait (FOG) occurs in 30–60% of the PD patients ‎[3]. Serious damage to multiple neuronal systems causing complex biochemical changes explained the severity of PD. Through the study of the PD pathology, researchers have put forward a variety of effective detecting PD methods, including resting state functional magnetic resonance imaging and the EEG signal analysis. However, on the one hand, the accuracy of detection rate is still not satisfactory, and, on the other hand, the methods used are complex and not efficient in detecting PD.

The German psychiatrist, Hans Berger, recorded the electrical currents in the brain which was discovered by Richard Canton, and he named them the electroencephalogram (EEG) ‎[4]. The clinical EEG signal is a time series of electrical potentials representing the sum of a very large number of neuronal dendritic potentials in the brain. It contains a lot of useful information relating to the different physiological states of the brain. In other words, the EEG signal is a useful way in understanding the complex dynamical behavior of the brain. Since EEG signal can be in a noninvasive way to record a long time spacing, we can exclude contingency and the monitoring incidental disorders under some conditions.

EEG records the brain electrical signals, and PD is a central nervous system dementia in which EEG signal abnormalities are more frequently shown ‎[5]. For these reasons, it is necessary to promote EEG signal detection techniques that help to identify PD clinically. In 2012, a method for detection of freezing of gait (FOG) in PD patients using EEG signals has been proposed by Handojoseno et al. ‎[6]. FOG is one of the symptom in PD patients; and their method used discrete wavelet transform (DWT) to decompose the EEG signal into EEG subbands, Wavelet Energy and Total Wavelet Entropy. Then, the features of PD patients' EEG signal would be identified by the backpropagation neural network classifier, and the accuracy is around 75%. Later on, they found that the frequency domain information was better than time domain information in discrimination EEG signals. In this case, they used the two domains in PD detection, acquiring the combination accuracy of 80.2%  [7]. Hansen et al. researched the idiopathic rapid eye movement (REM) sleep behavior disorder (iRBD) in early prediction of Parkinson's disease ‎[8]. They classified the subjects into two classes by the EEG similarity. The classical* K*-means and Bayesian classifier were the classification. The study demonstrated that the sensitivity and specificity will reach 80% and 90% from the classifiers above. Another two novel and effective methods were shown in the next. One of the methods is to characterize alterations in directional brain connectivity unique to PD with depression using the resting state functional magnetic resonance imaging (rs-fMRI) ‎[9]. Another method is, though, the changes of amplitude of low-frequency fluctuations (ALFF) in patients with PD ‎[10], and the rs-fMRI has been considered for development as a biomarker and analytical tool for evaluation of PD ‎[11]. The proposed methods could detect the significant alterations of ALFF in the subcortical regions and prefrontal cortex in PD patients.

Few studies have been applied EEG in PD patients; they found differences in the EEG signals recorded from PD patients and normal subjects. To make the detection method developed in this study much more fit to people's decision-making rules, three-way decision model was proposed in rough set theory, and it is very suitable in dealing with decision or classifying issue. In this study, we proposed a new combination scheme to detect the PD EEG signal in normal subject and PD patients. The discrete wavelet transform (DWT) decomposed the EEG into subbands. Then, using the sample entropy (SampEn), we analyzed these subbands and obtained the SampEn values. At last, the three-way decision model based on optimal center constructive covering algorithm (O_CCA) was designed to detect the EEG signal. The O_CCA was a classifier in the scheme, and it used the SampEn values as its condition attributes in classification. In the experiment, it is shown that the scheme we proposed is able to detect the PD EEG signal with a high accuracy.

This paper is organized as follows.  Section 2 describes the data acquirement and its format that we used in this work, describes the combination methods, respectively, gives the features that are in subbands decomposed by DWT, then describes SampEn method and SampEn values in detail (these methods aim to extract the features from EEG signal), and defines the attributes and O_CCA classifier that were used.  Section 3 presents the classification results, reviews the scheme, and discusses the results obtained in this study. In the end, Section 4 gives the conclusions.

## 2. Materials and Methods

### 2.1. Data

This study is designed according to the principles of the Declaration of Helsinki and approved by the Ethics Committee of the Second People's Hospital of Shenzhen in China. The EEG data are from 42 participants aged 63 to 78 years. All participants had been notified that we will take their EEG signal recording in this study. The participants include two cases: 25 healthy subjects that include 15 male (aged 65 to 73) and 10 female (aged 63 to 74) and 17 PD patients that include 10 male (aged 68 to 76) and 7 female (aged 70 to 78). All the participants do not have other diseases (such as insomnia, heart disease, and chronic obstructive pulmonary disease) which influence EEG signal in this study. The experiment of this study was performed in Matlab (R2014b, 64 bits).

**Figure 1:**
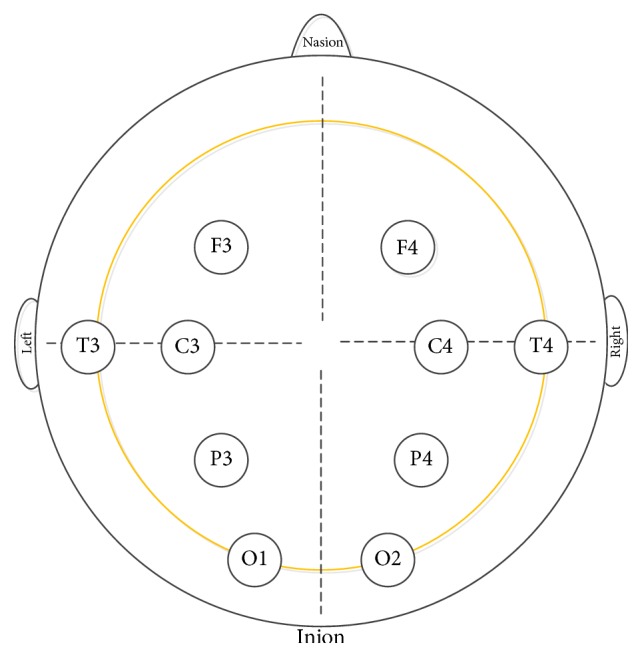
10-channel EEG electrode placement.

The clinical EEG signal data we used in this study is recorded by 10-channel EEG time series, and the recordings were made under awake and relaxed conditions. All subjects closed their eyes to reduce the nictation noise interference, and each recording continues 40 seconds at a sampling rate of 250 Hz. The 10-channel EEG electrode placement was shown in Figure 1, F3 and F4 channels were measured on the frontal region, C3 and C4 channels on the central region, T3 and T4 channels on the temporal region, P3 and P4 channels on the parietal region, and O1 and O2 channels on the occipital region.

### 2.2. Methodology

**Figure 2:**
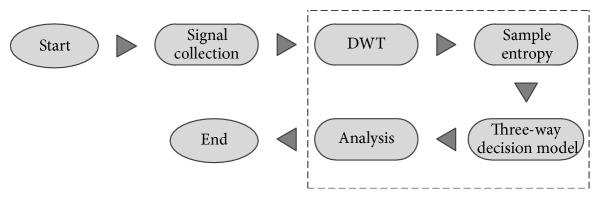
Overall EEG signals analysis procedure.

In this work, the EEG signals analysis procedures are composed of four main steps, boxed in Figure 2. We first apply DWT to decompose the EEG signal into subbands and then analyze these subbands which was significant part and compute the sample entropy. Use the SampEn as condition attributes in three-way decision model based on the optimal center constructive covering algorithm (O_CCA). At last, we use the three-way decision rules to analyze the EEG signal that belongs to PD patient or not. These methods are explained in detail below. DWT and SampEn methods are the feature extraction methods; three-way decision model is the classifier in this scheme, and also we compare the accuracy rate with other binary classifiers such as SVM and KNN in the end.

#### 2.2.1. Discrete Wavelet Transform

Wavelet transform (WT) and Fourier transform (FT) are widely applied to solve various problems in many research fields, respectively. The Fourier transform of a signal contains the frequency domain, but it lacks the time domain over the analysis window. The FT coefficient does not change with time and it is constant in the majority of situations ‎[12]. In this case, it cannot analyze the nonstationary signal with a minimum error. Besides, the FT coefficient is the global region and the time localization information cannot reflect very clearly. The short-time Fourier transform (STFT) is proposed to divide signal into sort time interval and then acquire time and frequency domain in the localization analysis windows. STFT could only give the fixed time and frequency analysis window with the entire signal. Over the past few decades, wavelet transform analysis has been developed as an improvement on Fourier transform especially in the STFT. Its main advantage in analyzing physiological systems is its capability to detect and analyze nonstationarity in signals and its related aspect like trends, breakdown points, and discontinuity. Wavelets are well localized in both time and frequency domain ‎[13]. In WT, we could get better low-frequency information in long time window and get high frequency in short-time window. Unlike Fourier transform, WT could generate sensitivity frequency information in a low frequency and provide sensitivity time detailed information in a high frequency.

Contrary to Fourier transforms, the continuous wavelet transform (CWT) ‎[14] on the basis of wavelet function *ψ*, scaled (*a*) and shifted (*b*) and the signal *x*(*t*), is defined by

(1) $$\begin{array}{c} CWT\left(\;a,b\;\right)=\overset{\infty }{\underset{-\infty }{\int }}x\left(\;t\;\right)\frac{1}{\sqrt{\left|a\right|}}\psi^{*}\left(\;\frac{t-b}{a}\;\right)dt, \end{array}$$

where (*∗*) denotes the complex conjugation, *a* is called scaling or reciprocal of frequency parameters, and *b* is called shifting or time localization parameters. The parameters of CWT were complicated in calculate. Therefore, the more efficient and brief wavelet analysis was present with the changed time scaled parameters (*a*, *b*), so that the correlation called discrete wavelet transform (DWT), such wavelet analysis, could be written as

(2) $$\begin{array}{c} DWT\left(\;j,k\;\right)=\frac{1}{\sqrt{\left|2^{j}\right|}}\overset{\infty }{\underset{-\infty }{\int }}x\left(\;t\;\right)\psi \left(\;\frac{t-2^{j}k}{2^{j}}\;\right)dt, \end{array}$$

with 2^*j*^ replacing *a* and 2^*j*^*k* replacing *b*. In the multiresolution signal analysis, Mallat proposed efficient methods to analyze the signal through a series of quadrature mirror filters which contains high-pass (HP) and low-pass (LP) filter.

**Figure 3:**
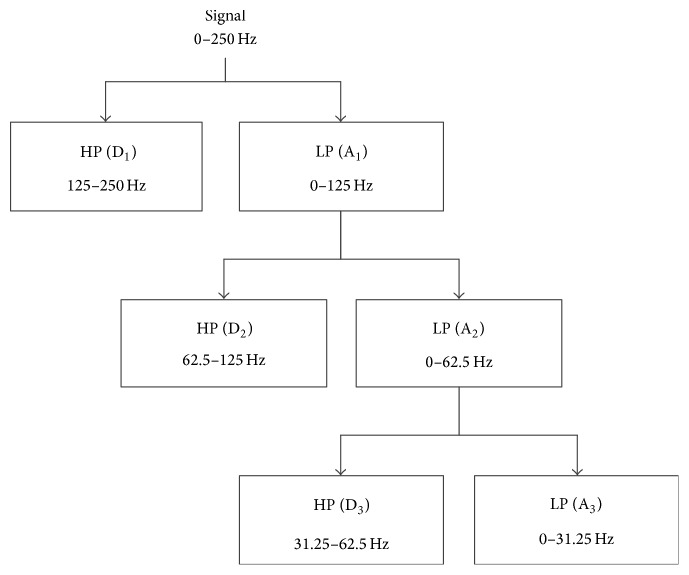
The structure of the three-level DWT of EEG signal.

In DWT algorithm, the signal passed through HP and LP filters to acquire the split frequency information ‎[15]. According to the basic theory of Nyquist sampling theorem ‎[16], the frequency information from the HP filter is referred to as detail coefficient; it has reserved the high frequency bandwidth of the original signal. Similarly, the frequency information from the LP filter is referred to as approximation coefficient; it has reserved the low-frequency bandwidth of the original signal. After the first-level filter, the detail and approximation coefficients are called D_1_ and A_1_, respectively. The structure of this wavelet decomposition with the corresponding EEG signal and its approximation and detail coefficients at each level are shown in Figure 3.

**Figure 4:**
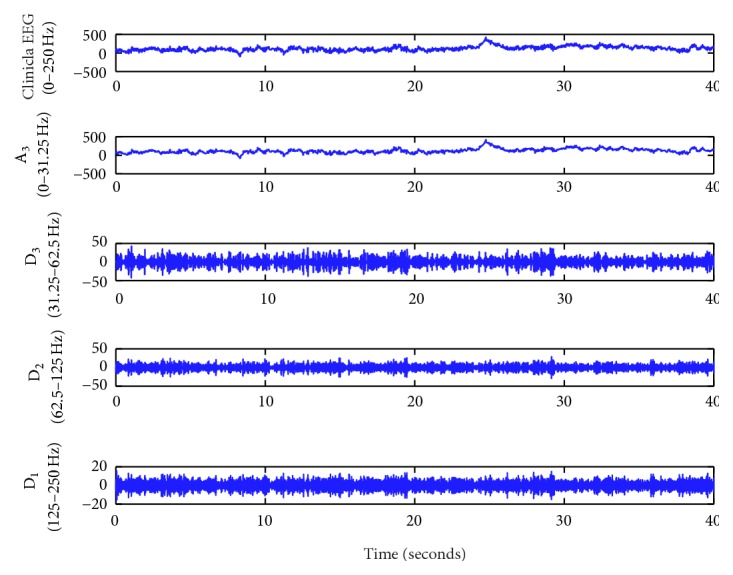
Applied three-level DWT of a clinical EEG signal.

In this study, DWT is the first stage in feature extraction. As the EEG signals have been noised by the movements of eyes and contractions of facial muscles in collecting process, we employed the three-level DWT to analyze EEG signal of both normal and PD patients ‎[17]. D_1_, A_1_, D_2_, A_2_, D_3_, and A_3_ represent each level detail and approximation coefficients. Here, we used *f* to represent the sampling frequency 250 Hz of original EEG signal, and the corresponding frequency bands of each detail and approximation coefficients are *f*/2 − *f*, 0 − *f*/2, *f*/4 − *f*/2, 0 − *f*/4, *f*/8 − *f*/4, 0 − *f*/8. The original EEG signal and its detail and approximation coefficients that applied the three-level DWT are shown in Figure 4. It has illustrated the amplitude and frequency alterations of the normal subject C4 channel of the signal.

#### 2.2.2. Sample Entropy

SampEn is a measure that quantifies the regularity or predictability of experimental EEG and biological time series. It is also a parameter to analyze the time series ‎[18]. SampEn was proposed by Richman and Moorman ‎[19]; it is a measure of signal regularity and complexity like approximate entropy (ApEn). The sample and approximate entropy both measure the data regularity of the data patterns of each other. Pincus proposed the ApEn algorithm ‎[20]; it is a stability and independent model. The detail of computing ApEn is shown in the following. Consider the time series of EEG signal, *x*(*n*), *n* = 1,2,…, *N*. A data pattern length *m*, which is also called embedding dimension, is used to construct the state vectors *u*(*i*) in the embedding space *R*^*m*^, *u*(*i*) = [*x*(*i*), *x*(*i* + 1),…, *x*(*i* + *m* − 1)], for each *i*, 1 ≤ *i* ≤ *N* − *m* + 1, and next

(3) $$\begin{array}{c} C_{i}^{m}\left(\;r\;\right)=\frac{1}{N-m+1}\overset{N-m+1}{\underset{j=1}{\sum }}\theta \left(\;r-d\left(\;u\left(\;i\;\right),u\left(\;j\;\right)\;\right)\;\right), \end{array}$$

with *θ* is the Heaviside function, *θ*(*x*) = 1 if *x* ≥ 0 and *θ*(*x*) = 0; otherwise, *r* is the signal comparison distance vector and *d*(*u*(*i*), *u*(*j*)) is distance measure written as

(4) $$\begin{array}{c} d\left(\;u\left(\;i\;\right),u\left(\;j\;\right)\;\right)=\underset{k=1,2,\ldots ,m}{\max}\left(\;\left|\;x\left(\;i+k\;\right)-x\left(\;j+k\;\right)\;\right|\;\right), \end{array}$$

with 1 ≤ *k* ≤ *m* − 1; 1 ≤ *i*, *j* ≤ *N* − *m* + 1,  *i* ≠ *j*. Then, define Φ^*m*^(*r*) as

(5) $$\begin{array}{c} \Phi^{m}\left(\;r\;\right)=\frac{1}{N-m+1}\overset{N-m+1}{\underset{i=1}{\sum }}\log C_{i}^{m}\left(\;r\;\right). \end{array}$$

We have the ApEn formulation defined by

(6) $$\begin{array}{c} ApEn\left(\;m,r,N\;\right)=\Phi^{m}\left(\;r\;\right)-\Phi^{m+1}\left(\;r\;\right), \end{array}$$

where the ApEn basically measures the logarithmic likelihood that runs of patterns close to length *m* within the same tolerance within *r* which will remain close on next incremental comparisons. To compute the ApEn, we should specify the two input parameters, *m* and *r*; it is also set to fixed values.

SampEn is largely independent of record length and displays relative consistencies under circumstances where ApEn does not ‎[21]. SampEn is an improved algorithm based on ApEn, which is a novel measure that quantifies the regularity or predictability of a time series. To calculate the SampEn, there is some similarity to ApEn, as the time series of data is *x*(*n*), *n* = 1,2,…, *N*, the other condition and *d*(*u*(*i*), *u*(*j*)) are similar to ApEn algorithm, and, next, for each *i*, 1 ≤ *i* ≤ *N* − *m*,  *i* ≠ *j*,

(7) $$\begin{array}{c} B^{m}\left(\;r\;\right)=\frac{1}{N-m}\overset{N-m}{\underset{i=1}{\sum }}\theta \left(\;r-d\left(\;u\left(\;i\;\right),u\left(\;j\;\right)\;\right)\;\right), \end{array}$$

and, for the same for the embedding dimension *M* = *m* + 1, we define *A*_*i*_^*M*^(*r*) as

(8) $$\begin{array}{c} A^{M}\left(\;r\;\right)=\frac{1}{N-m}\overset{N-m}{\underset{i=1}{\sum }}\theta \left(\;r-d\left(\;u_{M}\left(\;i\;\right),u_{M}\left(\;j\;\right)\;\right)\;\right), \end{array}$$

where

(9) $$\begin{array}{c} d\left(\;u_{M}\left(\;i\;\right),u_{M}\left(\;j\;\right)\;\right)=\underset{k=1,2,\ldots ,m}{\max}\left(\;\left|\;x\left(\;i+k\;\right)-x\left(\;j+k\;\right)\;\right|\;\right), \end{array}$$

with 1 ≤ *k* ≤ *M* − 1; 1 ≤ *i*, *j* ≤ *N* − *M* + 1, *i* ≠ *j*.

For fixed *m*, *r*, SampEn is given by the following formula:

(10) $$\begin{array}{c} SampEn\left(\;m,r,N\;\right)=-\ln\left[\;\frac{A^{M}\left(r\right)}{B^{m}\left(r\right)}\;\right], \end{array}$$

where *m* and *r* are the two input parameters, *m* means the length of time series that will be compared, and *r* represents the similarity criterion, and *N* is the length of the time series. It is imperative that parameters should be carefully chosen; in this study, we have calculated SampEn (*m*, *r*, *N*) for all signals with *m* = 2 and *r* = 20% of the standard deviation of the specific individual subject's time series ‎[22] and *N* as 10000. The signal length of each channel is fixed value.

Comparing (6) and (10), there are two major differences between ApEn and SampEn algorithm; SampEn does not statistics self-matches and does not use a template-wise approach when estimating conditional probabilities. It also shown that SampEn algorithm is of much less space complexity and time complexity. Hence, in this work, we use SampEn instead of ApEn to analyze the complexity and regularity of EEG signal in each channel. Then, the SampEn features were fed to the classifier three-way decisions model.

#### 2.2.3. Three-Way Decisions Model

**Figure 5:**
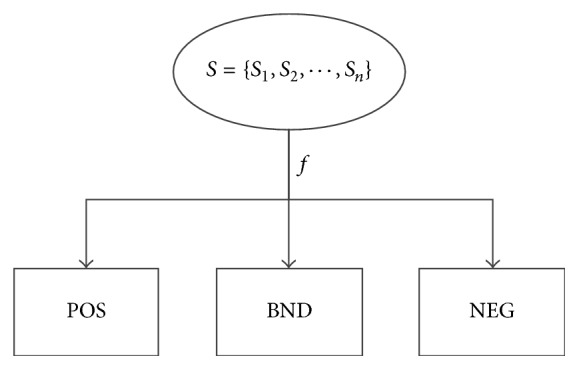
Three-way decisions model.

The theory of three-way decisions is proposed by Yao and used to interpret the three regions in rough set ‎[23], and it is constructed based on the notions of acceptance, rejection, and noncommitment ‎[24]. It is an extension of the traditional used binary-decision model with an added third option. The model is widely used in the uncertain or incomplete information areas, commonly used in everyday life, and widely applied in many fields. Based on the thresholds, it divides the universe into three regions as positive region (POS), boundary region (BND), and negative region (NEG) ‎[25], as shown in Figure 5. However, a challenge of the early study of three-way decision model based on the rough set, such as Decision Theoretic of Rough Set Model (DTRSM), is how to compute the thresholds ‎[26]. In most cases, the thresholds are calculated from given loss functions based on the experience of experts. In this case, the model cannot avoid artificial disturbance in selection of loss functions, as they could lead to a subjective result. In this paper, we applied the three-way decision model in detecting PD patients by the EEG features. So we should make the data analysis in objective way and decrease artificial disturbance.

We propose a novel three-way decision model based on the optimal center in constructive covering algorithm (O_CCA). It is the development of the CCA by exactly finding out the center of the covering regions ‎[27]. The O_CCA could produce three regions automatically according to the data; it not necessary to give parameters by the a priori knowledge. We classify the data based on the three regions. The realization steps of model based on O_CCA were introduced in the following.

We begin from the work by giving the data set *U* = {(*x*_1_, *y*_1_), (*x*_2_, *y*_2_),…, (*x*_*p*_, *y*_*p*_)}, where *x*_*i*_ = (*x*_*i*_^1^, *x*_*i*_^2^,…, *x*_*i*_^*n*^), (*i* = 1,2,…, *p*) represents the dimensional condition attribute of the sample and *y*_*i*_ is the decision attribute.

The first step of processing data is normalization, and then map data to (*n* + 1) dimensional sphere *S*^*n*+1^ by *T* : *U* → *S*^*n*+1^, $T(x)=(x,\sqrt{R^{2}-|x{|}^{2}})$, where *R* ≥ max⁡{|*x* | , *x* ∈ *U*}. We project the samples onto *n* + 1-dimensional sphere to make the length of each of the samples equal. And, then, we divided samples to training and testing based on the 10-fold cross-validation. For the training samples, we first select all the not covered samples belonging to the same class to constitute covering sets *C*_set_ and then compute *x*^mean^ from condition attributes. According to the nearest mean ‎[28] and Euclidean distance, we select the most closest sample *x*_*i*_ to *x*^mean^ from the *C*_set_ as the center of a cover. Compute the cover radius *θ*  [29]. Based on the center *x*_*i*_ and radius *θ*, get the covering sets *C* on *S*^*n*+1^. Execute the steps above until all the training samples are covered. At the end, we acquire a set of covers *C* = {*C*_1_, *C*_2_,…, *C*_*m*_}, where *C*_*i*_ = {*C*_*i*_^1^, *C*_*i*_^2^,…, *C*_*i*_^*m*_*i*_^}, representing that cover *C*_*i*_ has at least one subcover and includes all of the *i*th training samples.

According to the definition of three regions ‎[30] based on O_CCA, we can make three-way decisions on the test samples. We assume (*c*_1_^*k*^, *θ*_1_^*k*^) as the center and radius of *C*_1_^*k*^ ∈ *C*_1_, (*c*_2_^*l*^, *θ*_2_^*l*^) is the center and radius of *C*_2_^*l*^ ∈ *C*_2_. The rules of three-way decision of the test sample *x*_*t*_ are shown as follows:

1. *x*_*t*_ ∈ POS (*C*_1_), where the condition is dist (*x*_*t*_, *c*_1_^*k*^) ≤ *θ*_1_^*k*^ and dist (*x*_*t*_, *c*_2_^*l*^) > *θ*_2_^*l*^, meaning that *x*_*t*_ has the same decision attribute with the cover sets *C*_1_, and we have the right classification.
2. *x*_*t*_ ∈ NEG (*C*_1_), where the condition is dist (*x*_*t*_, *c*_1_^*k*^) > *θ*_1_^*k*^ and dist (*x*_*t*_, *c*_2_^*l*^) ≤ *θ*_2_^*l*^, meaning that *x*_*t*_ has the different decision attribute with the cover sets *C*_1_, and we did not classify it correctly.
3. *x*_*t*_ ∈ BND (*C*_1_), otherwise, it means that, under the judgment condition of the current, we cannot give its category directly. We need much more evidence to classify the samples in this cover.

**Figure 6:**
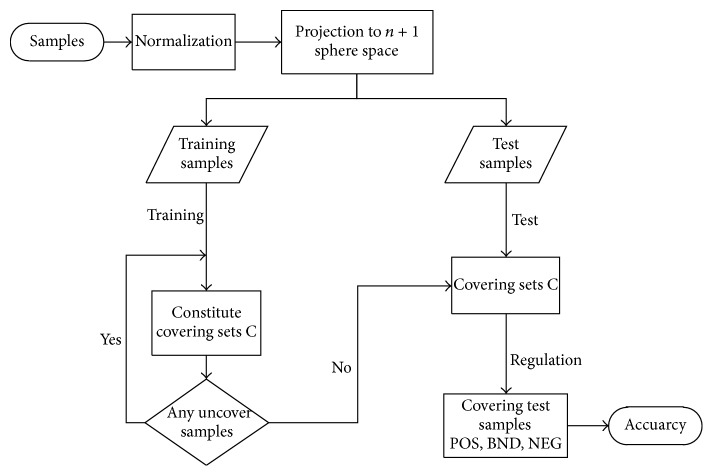
The O_CCA algorithm.

All the steps could be got from Figure 6 briefly. In this model, the three regions are formed automatically based on the distribution of the samples without any parameters. At the end, according to the decision rules, we can classify the test samples directly.

## 3. Result and Discussion

Detecting the PD patients' EEG signal from normal subjects and PD patients is a hard task for a trained professional visually. In our study, we used the DWT as the preprocessing step, as it provides the time-frequency localization information of signal and then uses sample entropy theory to acquire the SampEn of each of the channels. At last, we use the three-way decision model based on the optimal center constructive covering algorithm (O_CCA) as the classifier to distinguish the EEG signal.

Three-level DWT was applied to the clinical EEG signal for both normal subject and PD patients. The structure for this DWT along with the corresponding frequency bands of the approximations and detail coefficients at each level is in Figure 3. In Figure 4, the approximation and detail coefficients of channel C4 measured on the central region are shown.

**Figure 7:**
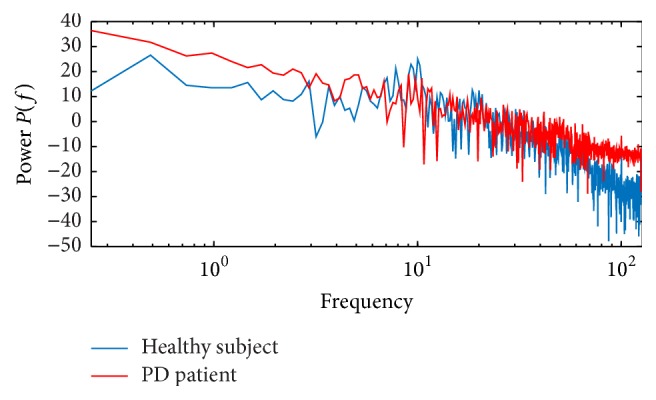
Power spectra (the occipital region O2-channel signal; the blue is healthy subject and red is PD patient).

Power spectra can provide information about the excitability of EEG signals ‎[31]. The composition of frequency of the different EEG frequency states is well-established.  Figure 7 shows the power spectra of channel O2 measured on the occipital region. Blue means the normal subject power spectra, and red means the power spectra of PD patient EEG signal. Comparing the two power spectra, a difference in the clinical EEG signal has been indicated, and it shows some understanding of the relationship between spectral information and SampEn. The significant difference of the two power spectra is in the 0–110 Hz, so the DWT of the EEG signal is necessary.

In the three-way decision model based on O_CCA, each instance used in the model contains 11 attributes, which consist of 10 condition attributes and one decision attribute. The 10 condition attributes are the SampEn of the 10 channels of one subject. The decision attributes are set by 0 to normal objects and 1 to PD patients. In order to detect the validity of the classification model, we have established three evaluation criteria, which are Acc = CCI/SI, Err = ECI/SI, Bnd = BI/SI, where SI means all EEG signal instances; CCI means the number of instances correctly classified in the POS regions; ECI means the number of instances mistakenly classified in the NEG regions; BI means the number of instances classified in the boundary regions. We compute the two forms of the data; they were originated in two types of SampEn, one that applied the DWT of clinical EEG signal.

The results of classifying subjects with three-way decision model based on O_CCA with two types of data are shown in Table 1. From the table, we can see that, in the same classifier, the data that applied DWT provided well performing results. The classification accuracy which applied DWT is 92.86%. It is higher than the accuracy 88.10% without DWT. This indicates that the DWT is an absolutely necessary part of analysis of EEG signal. Although applying DWT increases the complexity, the accuracy of classifier has improved obviously. We choose three-way decision model as classifier instead of the numerous binary classifiers; three-way decision model has boundary region compared to binary classifier. In detection of Parkinson's disease, the classifier we applied should not only have a high accuracy but also be suitable to analyze biomedical information. The instances in the boundary region mean delay decision; that is, by the present information, the model being unable to detect this instance is PD subject or normal. We need much more information to detect it. In the practical life, the boundary region represents the subject that needs further investigation.

To compare this classifier model efficiently, we use the applied DWT EEG data in the binary classifier and in common use binary classifier.  Table 2 shows that only classical SVM has higher accuracy than our model, but all these binary classifiers cannot provide the boundary region. It is arbitrary decision in diagnosis. Overall, the three-way decision model based on O_CCA has a high accuracy and gives a boundary region; these classification methods are much more suitable in detecting PD EEG signal than binary classifier. In this study, we have proposed a complexity analysis of EEG signal scheme, which combines three methods as follows: DWT, SampEn as the feature extraction methods, and O_CCA as the classifier method. As a combination scheme, DWT is necessary in clinical EEG signal preprocessing step; the study also demonstrated that choosing the SampEn for the classification condition attributes is sensible. In our study, we can infer from the classification accuracy that the three-way decision model based on O_CCA is suitable and efficient in analyzing the EEG signal.

In this study, in the feature, we need more subjects in order to generalize the results obtained in analysis. Simultaneously, we will abide by the standard of choosing participants, and we will also extract more data related to PD features in the following study. Possibly only some channels related to PD, not all channels, attribute reduction maybe improve the sensitivity in the detection of PD.

## 4. Conclusion

The clinical EEG signal can be used to detect normal subjects and PD patients by suitable methods. In this study, PD patients' EEG state detection has been proposed based on the DWT, sample entropy, and O_CCA. We have shown that the three-way decision model based on O_CCA via the SampEn as condition attributes could differentiate the EEG signal with clinically significant classification accuracy of 92.86. As a result of the experiment, the O_CCA is a suitable and efficient method in EEG signal analysis. Hence, the proposed scheme methodology is appropriate for the complexity analysis of the EEG dynamics in patients with Parkinson's disease.

## Figures and Tables

**Table 1:** Results of classification from the O_CCA classifier model.

EEG signal (SI)	DWT (yes/no)	Classifier	ECI	BI	CCI	Err (%)	Bnd (%)	Acc (%)
42	Yes	O_CCA	2	1	39	4.76	2.38	92.86
42	No	O_CCA	3	2	37	7.14	4.76	88.10

**Table 2:** The data applied DWT of results in binary classifier.

EEG signal (SI)	Classifier	ECI	CCI	Err (%)	Acc (%)
42	SVM	1	41	2.38	97.62
42	KNN	4	38	9.52	90.48
42	NB	5	37	11.90	88.10
42	RF	7	35	19.67	83.33
